# Designing Efficient Low-Cost Paper-Based Sensing Plasmonic Nanoplatforms

**DOI:** 10.3390/s18093035

**Published:** 2018-09-11

**Authors:** Laurentiu Susu, Andreea Campu, Ana Maria Craciun, Adriana Vulpoi, Simion Astilean, Monica Focsan

**Affiliations:** 1 Nanobiophotonics and Laser Microspectroscopy Center, Interdisciplinary Research Institute on Bio-Nano-Sciences, Babes-Bolyai University, Treboniu Laurean No. 42, Cluj-Napoca 400271, Romania; susulaurentiu@yahoo.com (L.S.); andreea.campu@gmail.com (A.C.); ana.gabudean@ubbcluj.ro (A.M.C.); simion.astilean@phys.ubbcluj.ro (S.A.); 2 Biomolecular Physics Department, Faculty of Physics, Babes-Bolyai University, M Kogalniceanu No. 1, Cluj-Napoca 400084, Romania; 3 Nanostructured Materials and Bio-Nano-Interfaces Center, Interdisciplinary Research Institute on Bio-Nano-Sciences, Babes-Bolyai University, Treboniu Laurian No. 42, Cluj-Napoca 400271, Romania; adriana.vulpoi@phys.ubbcluj.ro

**Keywords:** paper nanoplatform, gold nanorods, nanosensor, LSPR, SERS

## Abstract

Paper-based platforms can be a promising choice as portable sensors due to their low-cost and facile fabrication, ease of use, high sensitivity, specificity and flexibility. By combining the qualities of these 3D platforms with the optical properties of gold nanoparticles, it is possible to create efficient nanodevices with desired biosensing functionalities. In this work, we propose a new plasmonic paper-based dual localized surface plasmon resonance–surface-enhanced Raman scattering (LSPR-SERS) nanoplatform with improved detection abilities in terms of high sensitivity, uniformity and reproducibility. Specifically, colloidal gold nanorods (GNRs) with a well-controlled plasmonic response were firstly synthesized and validated as efficient dual LSPR-SERS nanosensors in solution using the p-aminothiophenol (p-ATP) analyte. GNRs were then efficiently immobilized onto the paper via the immersion approach, thus obtaining plasmonic nanoplatforms with a modulated LSPR response. The successful deposition of the nanoparticles onto the cellulose fibers was confirmed by LSPR measurements, which demonstrate the preserved plasmonic response after immobilization, as well as by dark-field microscopy and scanning electron microscopy investigations, which confirm their uniform distribution. Finally, a limit of detection for p-ATP as low as 10^−12^ M has been achieved by our developed SERS-based paper nanoplatform, proving that our optimized plasmonic paper-based biosensing design could be further considered as an excellent candidate for miniaturized biomedical applications.

## 1. Introduction

Over the last decade, there has been a significant increase in the development of portable biosensors which can be employed for rapid and inexpensive clinical analysis as diagnostics tools capable of disease screening, since an early diagnosis can change the evolution of the illness [1]. Unfortunately, the diagnostic procedures are complex, long and costly and the absence of properly equipped clinics is the biggest obstacle for the direct implementation of biosensors. Therefore, recent studies have been focused on developing simple, low-cost, efficient and accurate point-of-care (POC) platforms, thus providing rapid and portable biodetection of different analytes or biotargets of interest [2]. Notably, paper-based platforms have emerged as the best compromise between performance, cost and simplicity, becoming the perfect candidates as flexible substrates for the development of portable analytical devices, especially in the case of developing countries or resource-limited settings [3]. Such paper-based analytical devices have received a special interest due to their many advantages compared to plastic or glass solid substrates, such as their hydrophilic nature ensuring their ability to easily absorb fluids. More importantly, cellulose paper is a common biodegradable and biocompatible material [4], and all these features allow researchers to conduct new research and innovative applications, especially in the development of paper-based sensors with facile fabrication and operation strategies. As such, paper-based platforms can be applied in large areas such as nucleic acid detection, food quality control or clinical and environmental monitoring [5,6].

Remarkably, by integrating nanomaterials onto the cellulose fibers, it is possible to generate new functionalities of the paper platforms with improved properties. Currently, there are two general strategies to fabricate such nanomaterial-based analytical platforms: (i) by using pre-synthesized nanomaterials that will be immobilized on paper, or (ii) by directly performing an in situ synthesis of nanomaterials [7]. However, in order to design functional analytical platforms, a major challenge is to maintain the transduction ability of these immobilized nanoparticles. With this requirement in mind, one class of nanomaterials which, thanks to their unique optical properties, can improve the quality of the paper-based platforms [8] is represented by gold nanoparticles (GNP). In particular, these plasmonic NPs are attractive for optical detection, especially due to their strong absorption and scattering of light as well as the intense local electric field generation arising from localized surface plasmon resonance (LSPR) [9] relative to other metals. Additionally, the position of the LSPR band of GNPs depends on the shape, size, interparticle spacing and the local dielectric constant of the environment generated by the analyte found close to the NP [10]. On the other hand, the LSPR can be exploited to detect small target analyte molecules using surface-enhanced Raman scattering (SERS), as Raman scattering is significantly enhanced (typically by 104−108) when a target analyte is absorbed or present in the vicinity of the metal NP or rough metal surface [11,12], and furthermore, it can be used to specifically identify one or multiple analytes from complex structures. Best results are obtained using molecular structures which contain benzene rings, or amine or thiol groups that are highly susceptible to attaching to the metallic surface [13]. Although this surface-sensitive technique has continuously attracted a great deal of interest in the literature since 1977, the exact mechanism of the amplification effect is still in debate. However, two mechanisms are theoretically accepted: (i) the electromagnetic enhancement associated with the LSPR, and (ii) the chemical enhancement due to the charge transfer mechanism [14]. Therefore, it is important to design SERS platforms with strong signal enhancement and multiplex capabilities for the specific detection of biotargets.

Recently, our group has succeeded in fabricating a multifunctional sensing platform by integrating an LSPR transducer with SERS activity on a flexible three-dimensional (3D) Au nanocups substrate [15]. The same group successfully demonstrated the feasibility of gold bipyramidal-shaped nanoparticles (AuBPs) to be used as active plasmonic nanoplatforms for the detection of the biotin-streptavidin interaction in aqueous solution via both LSPR and SERS [16]. However, compared to other substrates, paper can yield better SERS enhancement due to its flexible three-dimensional (3D) matrix, onto which NPs can be easily adsorbed and stabilized [17]. More importantly, the paper substrate is beneficial not only as a 3D template but also for allowing capillary-driven flow without using external pumping; this inexpensive platform therefore being a “natural” microfluidic chip capable of further biological sensing [18]. In this context, Lee et al. reported a novel SERS substrate platform with high sensitivity to detect chemical traces such as trans-1,2-bis(4-pyridy)ethene (BPE) or 1,4-benzenedithiol (1,4-BDT) [19]. Recently, Ashley et al. developed an innovative SERS plasmonic paper platform via the in situ growth of anisotropic nanoparticles, a versatile approach that allows the improvement of the SERS performance, thus enabling the specific detection of serotonin, a molecular neurotransmitter with a weak affinity for Au [20]. The detection of the neuron-specific enolase in blood plasma, a traumatic brain injury protein biomarker, was also proved using a SERS-based paper platform [21].

Lately, there has been a great deal of interest towards anisotropic nanoparticles, such as gold nanorods (GNRs), as SERS-active nanosubstrates, which exhibit a locally enhanced electromagnetic field at their tips, thus generating strong intrinsic “hot spots” that do not need any additional aggregation approaches [22]. GNRs possess two well-resolved LSPR bands: one longitudinal band, parallel to the long axis of the rod, and one transversal band, perpendicular to the first [23]. Moreover, GNRs can absorb light from the visible to the near-IR region of the electromagnetic spectrum by chancing their aspect ratio, an important property that we should profit from to control and significantly improve SERS enhancement, especially by integrating them onto inexpensive paper substrates to provide a high degree of uniformity and reproductivity of the engineered large area plasmonic paper nanoplatform. For example, the Singamaneni group also developed excellent paper-based SERS substrates via the uniform decoration of common filter paper with plasmonic nanoparticles through immersion approach [24,25] or plasmonic calligraphy in order to obtain spatially isolated and multiplexed domains on the same platform [26,27].

Herein, we introduce a cheap, easy to fabricate, accurate, efficient dual LSPR-SERS nanoplatform based on immobilized GNRs with different aspect ratios on cellulose fibers of conventional filter paper. First, we evaluated the LSPR and SERS performance of synthesized GNRs with different aspect ratios in solution using p-aminothiophenol (p-ATP) as an active Raman reporter. The p-ATP molecule is extensively used in SERS analysis due to its unique Raman fingerprint; additionally, it attaches at the tips of the GNRs, which benefits both LSPR sensitivity and SERS activity [28]. As a result, we decided on the most efficient GNPs in terms of their LSPR and SERS sensitivity as a function of their longitudinal plasmonic band. Several methods are also available for paper patterning, such as inkjet or wax printing, but they are either expensive or require a complex fabrication process [29,30]. Next, we designed our plasmonic nanoplatforms by immobilizing a high concentration of GNRs with different aspect ratios on filter paper using a simple immersion approach. The as-obtained nanoplatforms were optically and morphologically characterized to understand their optical response and behavior. Then, the feasibility of the as-designed flexible paper-based plasmonic nanoplatforms to operate as both dual LSPR and SERS nanosensors was proved by combining the detection with the identification of p-ATP target analyte on the same nanoplatform. Specifically, their enhancement capabilities were evaluated by adding p-ATP analyte solutions with concentrations ranging from 10^−4^ to 10^−12^ M onto the paper. To keep the setup easy to carry out, the LSRP and SERS measurements of our plasmonic paper substrates were recorded using portable UV-Vis and Raman spectrometers, as detailed in Section 2. Finally, to evaluate the performance of the plasmonic-based paper nanoplatforms as LSPR-SERS sensing transducers, we experimentally performed a systematic comparison of the LSPR and SERS detection capability and achieved a limit of detection (LoD) of p-ATP analyte towards 10^−7^, by monitoring the LSPR shift, and 10^−12^ M by evaluating the intensity of the Raman band located at 1585 cm^−1^.

## 2. Materials and Methods

### 2.1. Chemicals

Tetrachloroauric acid (HAuCl_4_•4H_2_O, 99.99%), cetyltrimethylammonium bromide (CTAB, 96%), L-ascorbic acid (C_6_H_8_O_6_, 99%), hydroquinone (C_6_H_6_O_2_, HQ, 99%), sodium borohydride (NaBH_4_, 99%), silver nitrate (AgNO_3_, 99%), p-aminothiophenol (p-ATP) and Whatman^®^ qualitative filter paper, Grade 1 (Whatman no. 1) were purchased from Sigma-Aldrich. All chemicals were used without further purification. Ultrapure water (resistivity ~18.2 MΩ) was used as the solvent throughout the experiments.

### 2.2. Colloidal GNRs Synthesis

Colloidal GNRs were synthesized using the seed-mediated growth approaches previously described by Nikoobakht et al. [31] for smaller nanoparticles and Vigderman and Zubarev [32] for GNRs with LSPR responses at higher wavelengths. Both methods consist of two steps: the preparation of the gold seeds and the growth of the rod-like shaped nanostructures (see Figure S1).

Small GNRs with an LSPR less than 900 nm were prepared by the addition of 12 to 24 μL of seed solution, which involves mixing 0.4 M CTAB surfactant with 1 mM HAuCl_4_ and freshly prepared ice-cold 10 mM NaBH_4_ reducing agent solution, to a growth solution containing 0.2 M CTAB, 1 mM HAuCl_4_, various amounts of 4 mM AgNO_3_ and 78.8 mM ascorbic acid reducing agent. All reactants were added during mild stirring at 400 RPM using a Raypa magnetic stirrer. The mixture was left undisturbed at room temperature until it stabilized; the growth mechanism ends after around 30 min of reacting time.

In the case of high-yield GNRs with LSPR responses at higher wavelengths, the seed solution was prepared by mixing 25 mM HAuCl_4_ with 0.1 M CTAB surfactant solution under mild stirring followed by the dropwise addition of a 4 °C cold 10 mM NaBH_4_ solution under vigorous stirring (around 1400 RPM). A constant volume of 300 μL seed solution was added to a mixture of 0.1 M CTAB surfactant, 25 mM HAuCl_4_, different volumes ranging from 60 to 95 μL of 20 mM AgNO_3_ and 0.5 M hydroquinone reducing agent under mild stirring at 400 RPM using a Raypa magnetic stirrer. The final solution was left undisturbed overnight at 45 °C.

The colloidal GNRs were washed by centrifugation at 14,000 RPM for 15 min to remove excess reactants while the GNRs were dispersed in ultrapure water. The purification step was performed twice for further use.

### 2.3. Raman Labelling Protocol

**Scheme 1:**
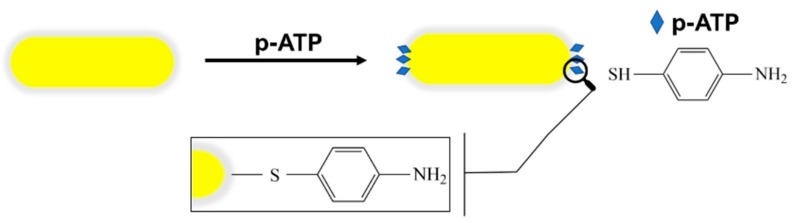
Schematic diagram illustrating the grafting of gold nanorods (GNRs) with p-aminothiophenol (p-ATP) analyte.

In order to determine the LSPR and SERS efficiency, 500 μL of the as-prepared colloidal GNRs with different aspect ratios were incubated with 10^−4^ M ethanoic p-ATP solution. The grafting protocol is schematically described in Scheme 1.

### 2.4. Fabrication of Paper-Based Plasmonic Nanoplatforms

Strips were cut from the paper sheet, immersed in the as-prepared GNRs solution until the saturation was reached and left to dry at room temperature. The p-ATP molecules were grafted onto the immobilized GNRs by dropwise wetting of the paper nanoplatform. To evaluate the LoD of the proposed plasmonic paper-based substrate via LSPR and SERS, different p-ATP concentrations spanning from 10^−4^ to 10^−12^ M were tested. It is important to note that, herein, the binding interaction of the p-ATP target and the plasmonic detection transducers occurs through 3D diffusion of the analyte molecules without any additional washing step, thus being closer to the much desired “no-wash biosensors”, which are particularly attractive for their direct, simple and rapid implementation of lab-on-paper devices [33].

### 2.5. Characterization Methods

The extinction spectra of the as-prepared GNRs were recorded using a Jasco V-670 UV-Vis-NIR spectrophotometer with a bandwidth of 2 and 1 nm spectral resolution. The size and morphology of the obtained GNRs were examined using a FEI Tecnai F20 field emission transmission electron microscope (TEM) operating at an accelerating voltage of 200 kV and equipped with Eagle 4k CCD camera. The colloid was added dropwise onto a carbon film-covered copper grid for TEM analyses and dried at room temperature. Dynamic light scattering (DLS) and Zeta Potential measurements were performed at 25 °C using a Zetasizer Nano ZS 90 from Malvern Instruments (Malvern, UK).

The optical properties of the paper-immobilized GNRs were determined using a portable Ocean Optics USB 4000 optical UV-Vis spectrophotometer equipped with an optical fiber with a core diameter of 600 μm. The morphology and the uniformity of the plasmonic-paper nanoplatforms were investigated by scanning electron microscopy (SEM) using a FEI Quanta 3D FEG scanning electron microscope operating at an accelerating voltage of 30 kV. Dark-field microscopy was performed using a ZEISS Axio Observer Z1 inverted microscope, equipped with a halogen lamp (HAL 100), a 20x ZEISS objective (NA = 1.4) and an achromatic–aplanatic immersion condenser (NA = 0.2–1.4).

The SERS spectra of both colloidal and immobilized GNRs were recorded with a portable spectrometer (Raman Systems R3000CN) equipped with a 785 nm diode laser coupled to a 100 μm optical fiber. The measurements were recorded at a set laser power of 200 mW and 10 s integration time per spectrum.

## 3. Results and Discussion

### 3.1. Optical and Morphological Characterizations of Colloidal GNRs

**Figure 1:**
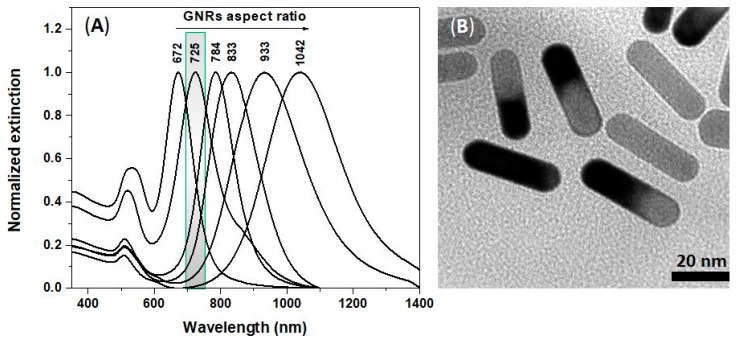
(**A**) Normalized UV-Vis-NIR extinction spectra of the synthetized GNRs in aqueous solution with different aspect ratios racing from 2.5 to 6.2; (**B**) representative TEM image illustrating the selected cetyltrimethylammonium bromide (CTAB)-stabilized GNRs at 725 nm.

First, the optical properties of the as-prepared GNRs in aqueous solution were determined. The normalized extinction spectra of colloidal GNRs with different aspect ratios are shown in Figure 1A. The two characteristic LSPR bands, both the transversal and longitudinal electron oscillation contributions, are present. While the transverse band is located at approximately 510 nm, the longitudinal LSPR band was tuned along the spectrum from 672 to 1042 nm and corelated with their aspect ratios ranging from 2.5 to 6.2. The fabrication of rod-like shaped nanoparticles with different aspect ratios is confirmed by the TEM investigations presented in the Supplementary Information in Figure S2. A representative TEM microscopic image of the GNRs at 725 nm is displayed in Figure 1B. The surface potential of the GNRs was investigated by zeta potential measurements and determined to be strongly positive, +42.5 mV (Figure S3B), validating the presence of the well-known positive CTAB surfactant bilayer on the surface of the GNRs [31].

### 3.2. LSPR-SERS Detection of p-ATP Analyte in Solution

In order to achieve the best performance of our tunable colloidal plasmonic NPs, the as-synthesized GNRs were investigated in terms of their optical response and electromagnetic enhancement in the presence of the well-known p-ATP Raman reporter for a better assessment of the LSPR and SERS efficiency. It is worth mentioning that we kept the used concentration of p-ATP, herein 10^−4^ M, constant for all tested colloidal GNRs. The p-ATP molecules interact strongly with the gold surface due to its thiols forming covalent bonds between the sulfur and Au atom via back π bonding, ensuring the successful attachment to the GNRs’ surface (see Scheme 1) [28]. Therefore, the normalized extinction spectra of the GNRs were recorded before and after the direct grafting of the p-ATP molecules on the metallic surface. As a first observation, no spectral broadening of the plasmonic bands was noticed. Moreover, the transversal band does not show any modification in position, while for the longitudinal LSPR response, red-shifts from 3 to 10 nm were noted in the presence of p-ATP molecules, indicating that they were successfully grafted on all GNRs systems. In fact, this red-shift of the longitudinal LSPR is due to the change of the surface refractive index, thus proving the capture of the p-ATP analyte at the exposed ends of the nanoparticles [16], which are more likely to attach molecules since the CTAB bilayer tends to bind more strongly to the {1 0 0} lateral faces and less to the end {1 1 1} and {1 1 0} crystal planes of the GNRs [33].

**Figure 2:**
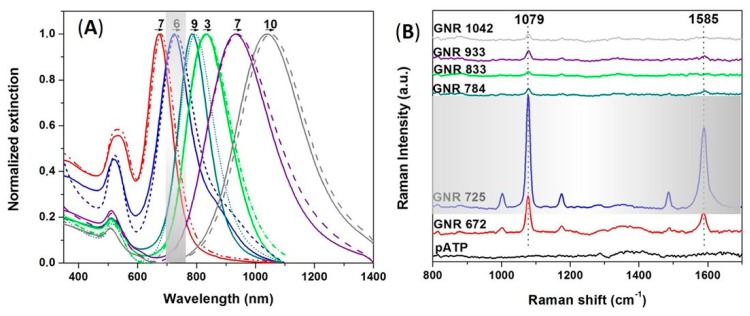
(**A**) Normalized extinction spectra of GNRs before (solid lines) and after (dotted lines) p-ATP grafting; (**B**) surface-enhanced Raman scattering (SERS) spectra of GNRs with different longitudinal localized surface plasmon resonance (LSPR) bands recorded after p-ATP grafting; the black spectrum represents the Raman spectrum of free p-ATP 10^−4^ M solution; Excitation laser line used: 785 nm.

Furthermore, to evaluate the maximum Raman amplification, the p-ATP@GNRs systems were examined by SERS spectroscopy by tailoring the longitudinal LSPR band relative to the laser excitation wavelength, herein using a portable laser excitation of 785 nm (Figure 2B). Compared to LSPR, the SERS technique provides molecular specificity and higher sensitivity than LSPR, allowing up to single molecular identification [34]. In the literature, the longitudinal LSPR band of the nanoparticle was typically placed near the laser excitation to generate a strong plasmon excitation and enhanced local field amplification [35]. In our case, while all the samples exhibit the characteristic p-ATP bands arising from the C–S and C–C stretching vibrations at 1079 cm^−1^ and 1585 cm^−1^, respectively [36], confirming along with the LSPR measurements the successful grafting of the analyte. Tiwari et al. systematically investigated the adsorption mechanism of aminothiophenol on the gold nanorods’ surface (both in solution or deposited on ITO substrate) using different excitation wavelengths [37]. We mention that no specific vibrational Raman bands have been detected from free p-ATP molecules at similar concentrations, as depicted in the black spectrum in Figure 2B. The most intense vibrational modes of p-ATP are shown by the GNRs with LSPR at 672 and 725 nm, the latter presenting the best SERS enhancement from all the samples (marked spectrum in Figure 2B). This result is in good agreement with the already-reported study proving that the strongest SERS enhancement is generated by NPs (in their case, Au nanostars) with a plasmonic response slightly blue-shifted from the laser excitation [38]. More specifically, the amplification of these already-mentioned two specific Raman bands, in our case, is explained by the electromagnetic effect, reflecting a vertical or tilted orientation of p-ATP molecules on the metallic surface [39] and being therefore a direct proof that the target analytes are covalently grafted on the two tips of GNRs. As such, the tips of the GNRs not only contribute as intrinsic hot spots that increase the sensitivity to the local modification of the refractive index, but also serve to promote efficient SERS identification and the detection of molecules of interest. To conclude, the GNRs with a longitudinal LSPR at 725 nm, blue-shifted compared to the excitation laser line, present the best SERS efficiency and a 6 nm red-shift of the LSPR band, being the best candidates of choice for further immobilization on the porous surface of the paper substrate.

Moreover, the stability of these nanosystems was investigated by repeating the SERS measurements after 24 h. Simply for exemplification, in Figure S4, the comparison between the SERS spectra of the selected GNRs with a plasmonic response at 672 nm recorded immediately and after 24 h of p-ATP grafting is presented. However, the recorded SERS spectra show an additional amplification of the characteristic p-ATP bands when compared to the initially recorded ones for all plasmonic nanosystems. This phenomenon can be explained by the high p-ATP concentration; even though molecules bind at the tips of the GNRs immediately after the addition, the grafting reaction is time-dependent, leading to the saturation of the reactive GNRs surface and then the removal of the CTAB molecules, thus decreasing the stability of the nanoparticles in solution and implicitly inducing a slight aggregation of the GNRs. The formation of aggregates favors the appearance of the so-called extrinsic SERS “hot spots”, generated from plasmon coupling, which induce a much higher local electromagnetic field in between GNR gaps [28].

### 3.3. Fabrication of Tunable Paper-Based Plasmonic Nanoplatforms

**Figure 3:**
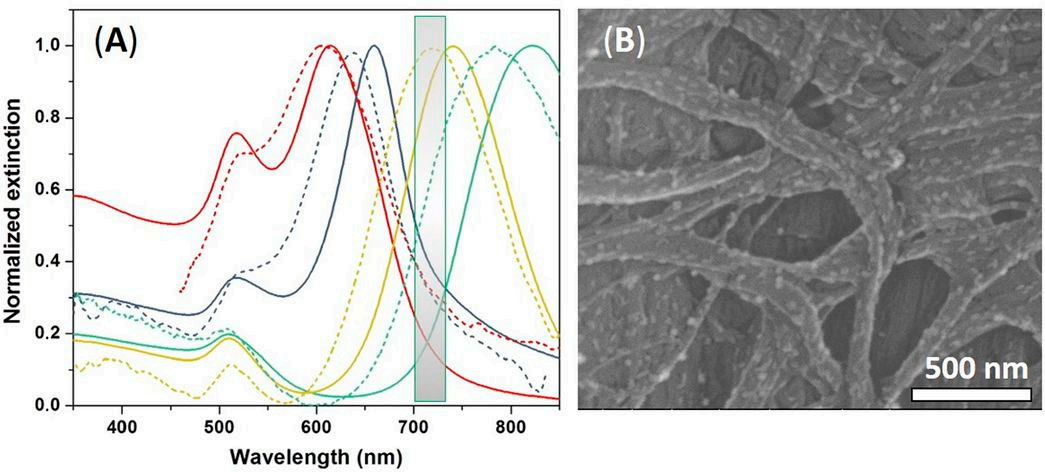
(**A**) Normalized extinction spectra of the GNRs with tunable LSPR response before (solid lines) and after the immobilization of the GNRs on the flexible paper platform (dotted lines); (**B**) representative SEM image of the plasmonic paper with a plasmonic response at 719 nm corresponding to the marked spectrum in Figure 3A.

For the fabrication of our plasmonic nanoplatform design, the paper used is common laboratory filter paper, Whatman no. 1, composed of α-cellulose (98%), ensuring negligible interference from components such as trace elements or coatings [19]. Microscale (~10 μm) cellulose fibrous stands are interweaved with smaller micro (average diameter of ~0.4 μm) and nanofibers. We also confirmed the hierarchical fibrous morphology of the filter paper by optical, SEM and dark-field investigations as presented in Figure S5A,C,D. However, after the immobilization protocol, the paper@GNRs nanoplatforms were optically characterized. Compared with the extinction spectra of the colloidal GNRs, our plasmonic nanoplatforms show the preserved typical optical response of the used GNRs (Figure 3A), the stationary transversal band at around 510 nm and the longitudinal LSPR band, which shows a blue-shift of 10 to 35 nm due to the effective refractive index decreasing from 1.333 (water) to 1 (air/substrate). This blue-shift was already reported in the literature by the Singamaneni group [24]. It is important to note that different values of recorded blue-shifts are due to the different refractive index sensitivities of the longitudinal LSPR [40]. Furthermore, it is worth mentioning that the density of the immobilized GNPs onto the paper fibers increases with the exposure time (data not shown); the extinction spectra presented in Figure 3A being recorded after their saturation have been reached.

In order to confirm the immobilization of the GNRs onto the paper substrates, representative SEM images were firstly acquired. Figure 3B and Figure S5B confirm the homogeneous absorption of the GNRs on the cellulose fibers; moreover, we observe the nanoparticles’ dense and uniform distribution on the fibers without visible large-scale aggregation. Specifically, the GNRs were efficiently immobilized onto the paper fibers via strong electrostatic interaction between the negatively charged cellulose, due to the hydroxyl groups on its surface [39,40] and the positively charged CTAB coated-GNRs. More importantly, we find that once the immobilization step of the GNRs on fibers paper was realized, even several times rinsing with water did not change the plasmonic GNR density, thus confirming the stability of our plasmonic-paper based nanoplatforms even after 2 months (data not shown).

Moreover, optical and dark-field images of the same samples stand in great agreement with the SEM measurements (Figure S5C–E). The observation of the immobilized nanoparticles using dark-field microscopy is possible due to the much larger scattering cross-section of the GNRs than the nanoparticle size [41]; the color of the metallic nanoparticles in dark-field microscopy is usually attributed to their scattering spectra [42], and therefore the investigated paper@GNRs with LSPR at 719 nm exhibit red scattering spots/lines due to the predominant longitudinal resonance (Figure S5E). Similarly, SEM, optical and dark-field images present high amounts of GNRs attached to the cellulose fibers (arrows on the images), confirming the successful fabrication of plasmonic nanoplatforms with a tunable LSPR response by a simple dip-coating of filter paper with different aspect ratio GNRs.

### 3.4. Evaluating the Tunable Paper-Based Nanoplatforms as Efficient Nanosensors

**Figure 4:**
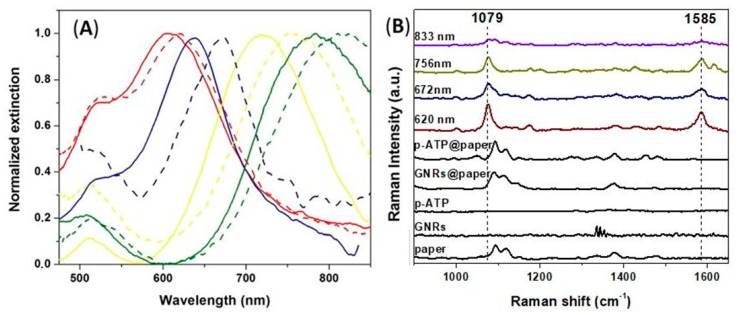
(**A**) Normalized extinction spectra of the GNRs immobilized onto the paper before (solid lines) and after (dotted lines) dropping the p-ATP analyte; (**B**) SERS spectra of the p-ATP molecules adsorbed onto fabricated tunable paper based plasmonic nanoplatforms; the black lines represent the Raman spectrum of Whatman paper No. 1., colloidal GNRs, p-ATP analyte alone (10^−4^ M), GNRs immobilized on paper, and free p-ATP 10^−4^ M dropped onto the bare paper, respectively; excitation laser line used: 785 nm.

Taking advantage of the facile tunability of the LSPR response of the paper-based nanoplatforms in order to obtain the maximization of the SERS signal, we next evaluate the SERS efficiency of the fabricated-plasmonic nanoplatforms, employing the same protocol, namely 10^−4^ M concentrated ethanoic p-ATP solution dropped onto the plasmonic nanoplatforms. As previously mentioned, GNRs are covered on their lateral faces by CTAB molecules, which bind electrostatically onto the cellulose fibers, leaving the exposed ends to interact with the p-ATP molecules. After the p-ATP is covalently bound to the previously immobilized GNRs, the longitudinal LSPR band is red-shifted, confirming the LSPR detection of the analyte (Figure 4A). The binding effect is highlighted by all the samples and translated from a surface refractive index change in the close vicinity of the GNRs. Specifically, the measured LSPR shift is related to the modification of the surface refractive index which is due to the capture of analyte at the nanoparticles’ surface at a specific active sensing area, in our case, the “hot spots” at the end of the individual GNRs, where the local electromagnetic field is stronger. However, excellent articles have explained this shift in LSPR frequency upon analyte binding in depth [43]. Notably, the presence of the analyte is first confirmed by the LSPR detection technique, the plasmon resonances’ red-shift providing high sensitivity (but not molecular specificity), while the specific identification of the target could be synergistically provided by SERS detection, which is able to offer molecular information about the analyte molecules found in the close proximity of the GNRs surface. For this reason, the SERS performance of these tunable paper-based plasmonic nanoplatforms was subsequently tested, employing a 785 nm excitation line. Figure 4B presents the recorded SERS spectra of the p-ATP molecules onto the as-fabricated tunable paper based plasmonic nanoplatforms together with their corresponding Raman spectra collected from bare paper (without GNPs), colloidal GNRs, p-ATP analyte alone (10^−4^ M), GNRs immobilized on paper, and p-ATP 10^−4^ M dropped onto the bare paper, respectively. As a result, all p-ATP@paper@GNRs substrates exhibit the two characteristic vibrational bands of p-ATP molecules located at 1079 cm^−1^ (C–S stretching) and 1585 cm^−1^ (C–C stretching) (Figure 4B), proving the specific SERS signature of the p-ATP molecules. It is important to note that the Raman fingerprint of the paper itself does not overlap/interfere with the p-ATP signature (see Figure 4B, black spectrum). Moreover, the SERS signal is more intense for the paper nanoplatforms with the immobilized GNRs exhibiting longitudinal LSPR bands at 620 nm and 756 nm, indicating the same behavior as in solution, which leads us to the conclusion that our plasmonic paper-based nanoplatform fabrication method well preserves the optical properties of the colloidal GNRs.

**Figure 5:**
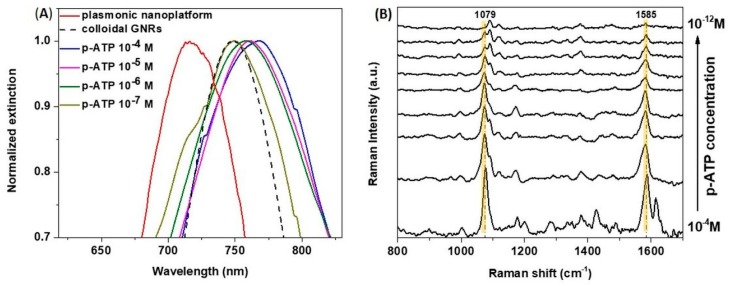
(**A**) Comparison between the position of the longitudinal LSPR bands corresponding to pure plasmonic nanoplatforms before and after adding different concentration of p-ATP molecules; (**B**) SERS spectra of the plasmonic nanoplatforms recorded at varying p-ATP concentration; excitation laser line used: 785 nm.

Finally, the sensitivity of our plasmonic nanoplatforms was experimentally evaluated by calculating the limit of detection (LoD) via LSPR and SERS measurements. The plasmonic nanoplatform used for this experiment exhibits the longitudinal LSPR band at 756 nm. Six microliters of p-ATP solution with concentrations ranging from 10^−4^ to 10^−12^ M were used for the evaluation of the optimized paper@GNR sensitivity. The LoD via LSPR detection was determined by measuring the LSPR red-shift as the difference between the optical response of the unfunctionalized and the p-ATP-grafted paper@GNRs substrate and reached the ethanoic p-ATP concentration of 10^−7^ M (Figure 5A and Figure S4A). In order to confirm the experimentally recorded LSPR red-shift of the plasmonic nanoplatform after the p-ATP grafting, we have performed finite-difference time-domain (FDTD)-based numerical simulations using the commercially available FDTD solutions software from Lumerical Inc. (Vancouver, BC, Canada) [44]. Concretely, the experimental position of the extinction bands is well reproduced by the FDTD simulated extinction spectrum presented in Figure S7, by considering the case of an individual GNR with 40 nm length and 12 nm diameter, estimated from TEM, in the numerical simulation. The refractive index values for water, gold and cellulose were taken from CRC data tables [45]. The GNR on cellulose (placed in air, *n* = 1) was modelled with a 4 nm CTAB coating of *n* = 1.435 and on the ends with an effective refractive index of 1.36. Subsequently, the p-ATP layer on the ends of the nanorod in water was modelled with an effective refractive index of 1.39, obtained from a 10% coverage with p-ATP (*n* = 1.66). As such, the FDTD-simulated optical response after p-ATP grafting is in good agreement with the experimentally recorded LSPR response of the paper-based nanoplatform. Subsequently, in the case of the SERS detection, the LoD was calculated by measuring the SERS intensity of the 1585 cm^−1^ peak as a function of p-ATP concentration (Figure 5B and Figure S4B). SERS has proven to be a more sensitive tool for quantitative detection at low concentrations, our design reaching a LoD of 10^−12^ M p-ATP analyte proving the high sensitivity of our paper-based sensing nanoplatforms.

## 4. Conclusions

In summary, we have developed a new flexible, portable, low-cost, easy to use and efficient dual LSPR-SERS plasmonic nanoplatform based on GNRs immobilized on common laboratory filter paper by a simple immersion procedure. Concretely, we synthesized tunable anisotropic rod-like nanoparticles with proven high LSPR spectral sensitivity and enhanced SERS activity due to the amplified electromagnetic field at their tips and took advantage of these optical properties to successfully fabricate plasmonic paper-based nanoplatforms with preserved capabilities. In this matter, we proved that our plasmonic substrate design is able to detect through LSPR up to a target analyte concentration of 10^−7^ M and to concomitantly identify it through SERS, proving its high sensitivity by achieving a LOD of 10^−12^ M. Our work suggests that the plasmonic paper-based nanoplatforms are an excellent choice for miniaturized optical biosensing applications due to their ease-of-use, flexibility, specificity and accuracy by employing low-cost materials and portable equipment.

## Supplementary Materials

The following are available online at http://www.mdpi.com/1424-8220/18/9/3035/s1, Figure S1: Schematic illustration of the seed-mediated method employed for the growth of GNRs. Figure S2: Representative TEM images of the selected GNRs with the longitudinal plasmonic band located at 672, 725, 784 and 833 nm, respectively, corresponding to the LSPR spectra presented in Figure 2A. Figure S3: (A) DLS and (B) Zeta potential data of GNRs with longitudinal LSPR band at 725 nm. Figure S4: Comparison between the SERS spectra of GNRs 672 recorded immediately and after 24 h of p-ATP grafting. Figure S5: Representative (A,B) SEM, (C,D) optical and (E,F) dark-field images of the bare paper platform and paper@GNRs nanoplatforms, respectively; Arrows indicate the GNRs immobilized on the paper fibers. Figure S6: LoD determined via (A) LSPR by measuring the LSPR shift of the p-ATP grafted paper nanoplatforms with different concentrations of p-ATP analyte and (B) SERS by measuring the SERS intensity of 1585 cm^−1^ band as function of p-ATP concentration onto the optimized plasmonic nanoplatforms. Figure S7: Experimental (solid) and FDTD simulated extinction (dotted) spectra obtained from the considered paper-based plasmonic nanoplatform before (red spectra) and after p-ATP grafting (blue spectra).
