# Supplementary material for: Designing Efficient Low-Cost Paper-Based Sensing Plasmonic Nanoplatforms
Source: Sensors (Basel). 2018 Sep 11;18(9):3035. doi: 10.3390/s18093035 (PMC6163559; doi:10.3390/s18093035)
Supplement: Supplementary file 1 [file sensors-18-03035-s001.zip › sensors-349518-supplementary.pdf]

sensors

MDPI

Supplementary Information

Designing Efficient Low-Cost Paper-based Sensing Plasmonic Nanoplatforms

Laurentiu Susu[1,†], Andreea Campu[1,2†], Ana Maria Craciun[1], Adriana Vulpoi[3], Simion Astilean[1,2] and Monica Focsan[1,*]

[1] Nanobiophotonics and Laser Microspectroscopy Center, Interdisciplinary Research Institute on Bio-Nano-Sciences, Babes-Bolyai University, Treboniu Laurean No. 42, Cluj-Napoca 400271, Romania; susulaurentiu@yahoo.com (L.S.) andreea.campu@gmail.com (A.C.); ana.gabudean@ubbcluj.ro (A.M.C) simion.astilean@phys.ubbcluj.ro (S.A.)

[2] Biomolecular Physics Department, Faculty of Physics, Babes-Bolyai University, M Kogalniceanu No. 1, Cluj-Napoca 400084, Romania;

[3] Nanostructured Materials and Bio-Nano-Interfaces Center, Interdisciplinary Research Institute on Bio-Nano-Sciences, Babes-Bolyai University, Treboniu Laurian No. 42, Cluj-Napoca 400271, Romania adriana.vulpoi@phys.ubbcluj.ro (A.V.)

* Correspondence: monica.iosin@phys.ubbcluj.ro (M.F.); Tel.: +40-264-45-45-54/116

† These authors contributed equally to this work

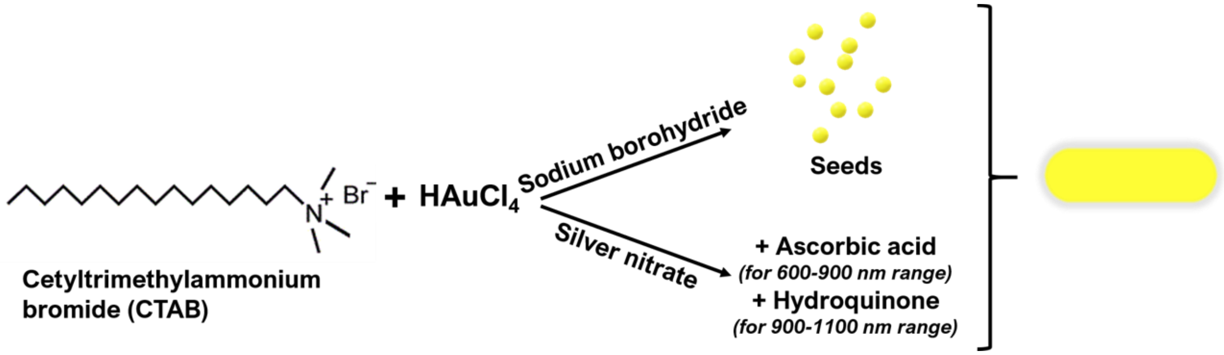

Figure S1. Schematic illustration of the seed-mediated method employed for the growth of GNRs.

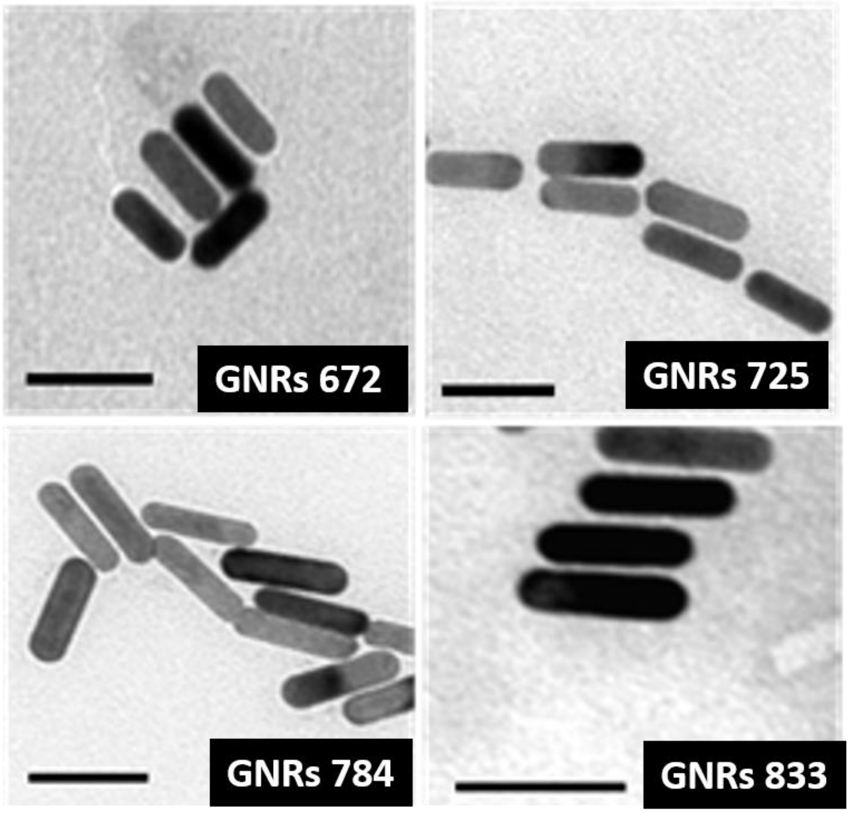

Figure S2. Representative TEM images of the selected GNRs with the longitudinal plasmonic band located at 672, 725, 784 and 833 nm, respectively, corresponding to the LSPR spectra presented in Figure 2A.

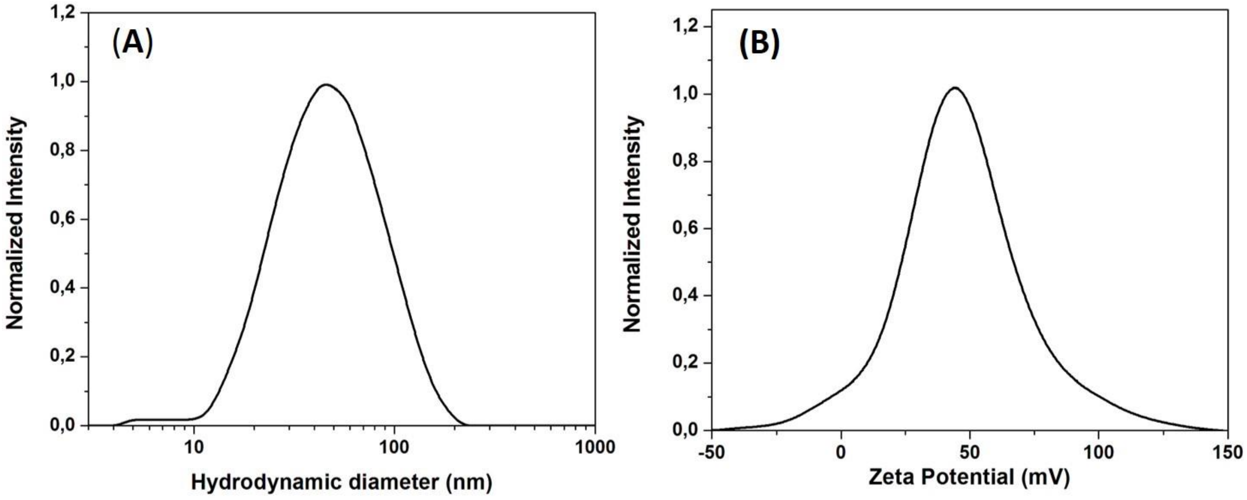

Figure S3. (A) DLS and (B) Zeta potential data of the selected GNRs with longitudinal LSPR band at 725 nm.

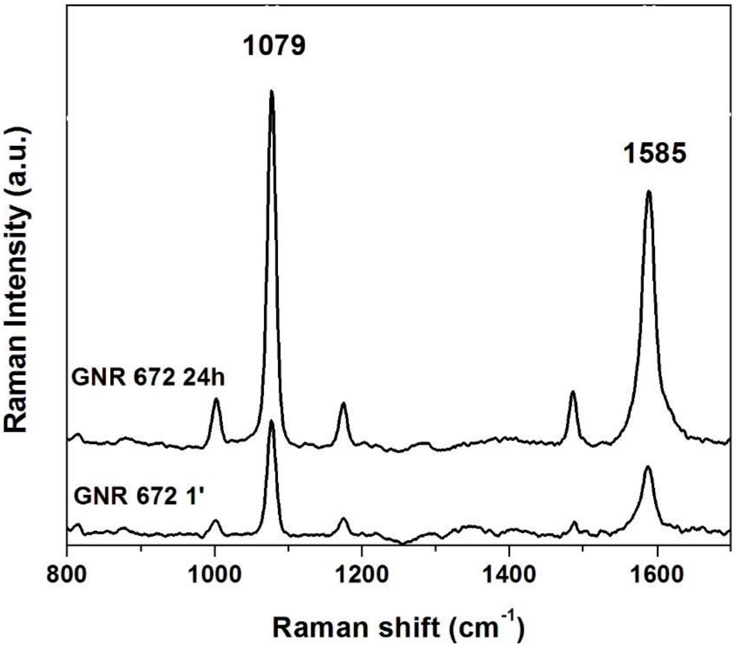

Figure S4. Comparison between the SERS spectra of GNRs 672 recorded immediately and after 24 hours of p-ATP grafting.

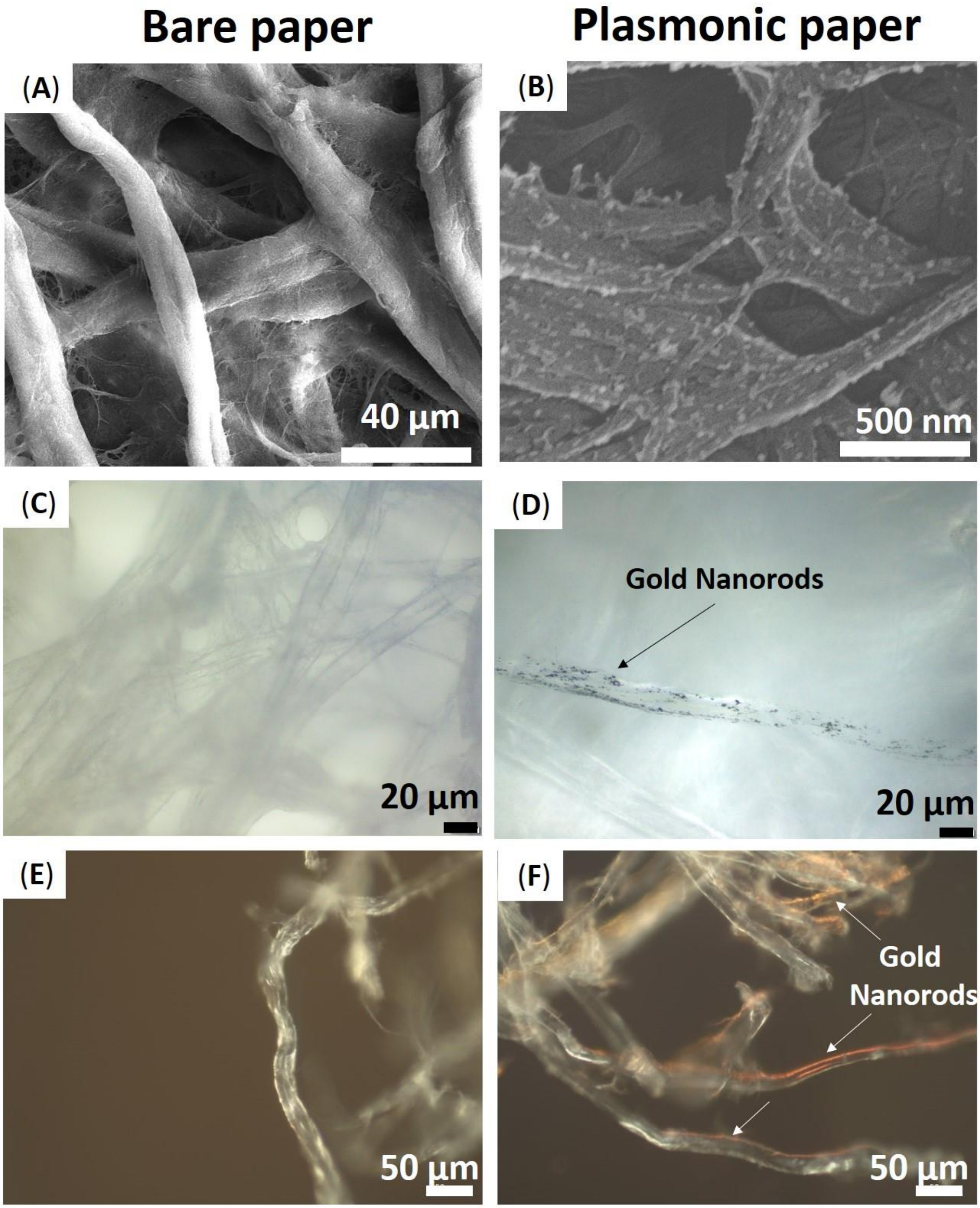

Figure S5. Representative (A, B) SEM, (C, D) optical and (E, F) dark-field images of the bare paper platform and paper@GNRs nanoplatforms, respectively; Arrows indicate the GNRs immobilized on the paper fibers.

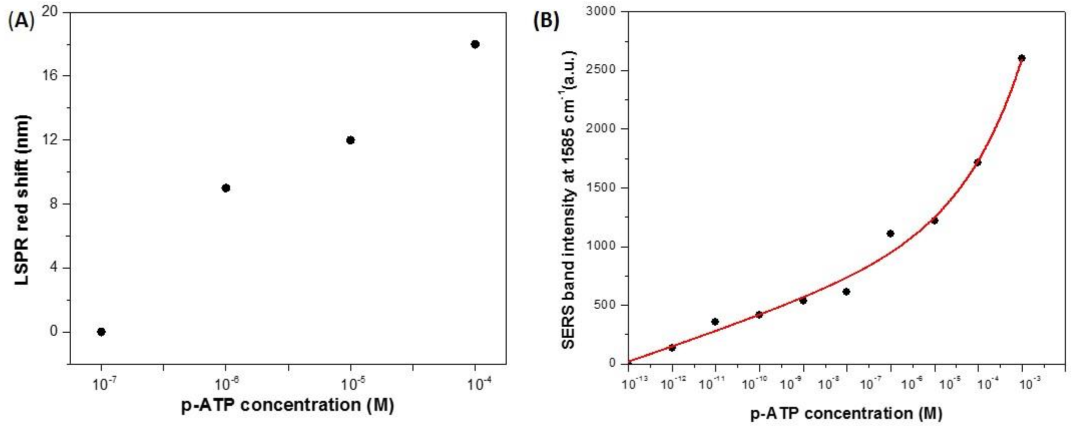

Figure S6. LoD determined *via* (A) LSPR by measuring the LSPR shift of the p-ATP grafted paper nanoplatforms with different concentrations of p-ATP analyte and (B) SERS by measuring the SERS intensity of 1585 cm^{-1} band as function of p-ATP concentration onto the optimized plasmonic nanoplatforms.

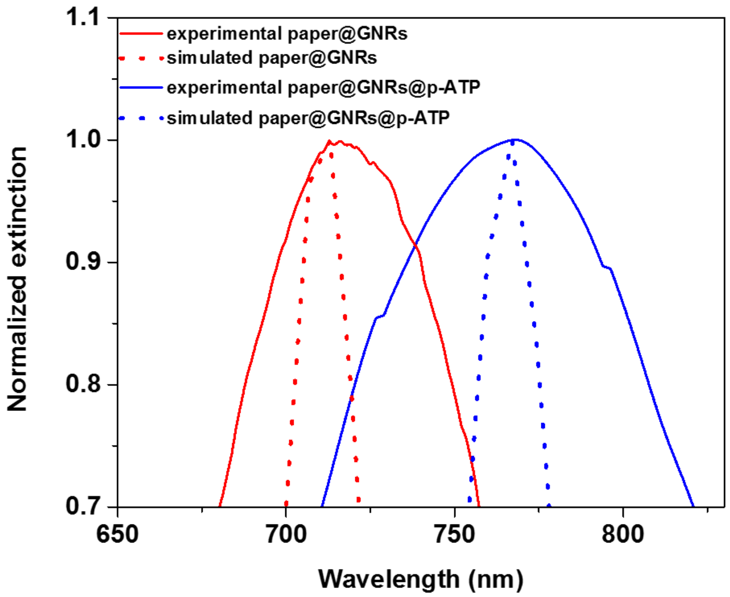

Figure S7. Experimental (solid) and FDTD simulated extinction (dotted) spectra obtained from the considered paper-based plasmonic nanoplatform before (red spectra) and after p-ATP grafting (blue spectra).
